# Supplementary material for: Dynamics of Hydrology and Anaerobic Hydrocarbon Degrader Communities in A Tar-Oil Contaminated Aquifer
Source: Microorganisms. 2019 Feb 9;7(2):46. doi: 10.3390/microorganisms7020046 (PMC6406676; doi:10.3390/microorganisms7020046)
Supplement: Supplementary file 1 [file microorganisms-07-00046-s001.pdf]

Supplementary Materials

Dynamics of Hydrology and Anaerobic Hydrocarbon Degrader Communities in a Tar-Oil contaminated Aquifer

Giovanni Pilloni[1], Anne Bayer[1], Bettina Ruth-Anneser[1], Lucas Fillinger[1], Marion Engel[2], Christian Griebler[1] and Tillmann Lueders[1,*]

[1] Institute of Groundwater Ecology, Helmholtz Zentrum München - German Research Center for Environmental Health, Neuherberg, Germany

[2] Research Unit Comparative Microbiome Analysis and Research Unit Scientific Computing, Helmholtz Zentrum München

* Correspondence: tillmann.lueders@uni-bayreuth.de. Present address: Department of Ecological Microbiology, University of Bayreuth, 95440 Bayreuth, Germany.

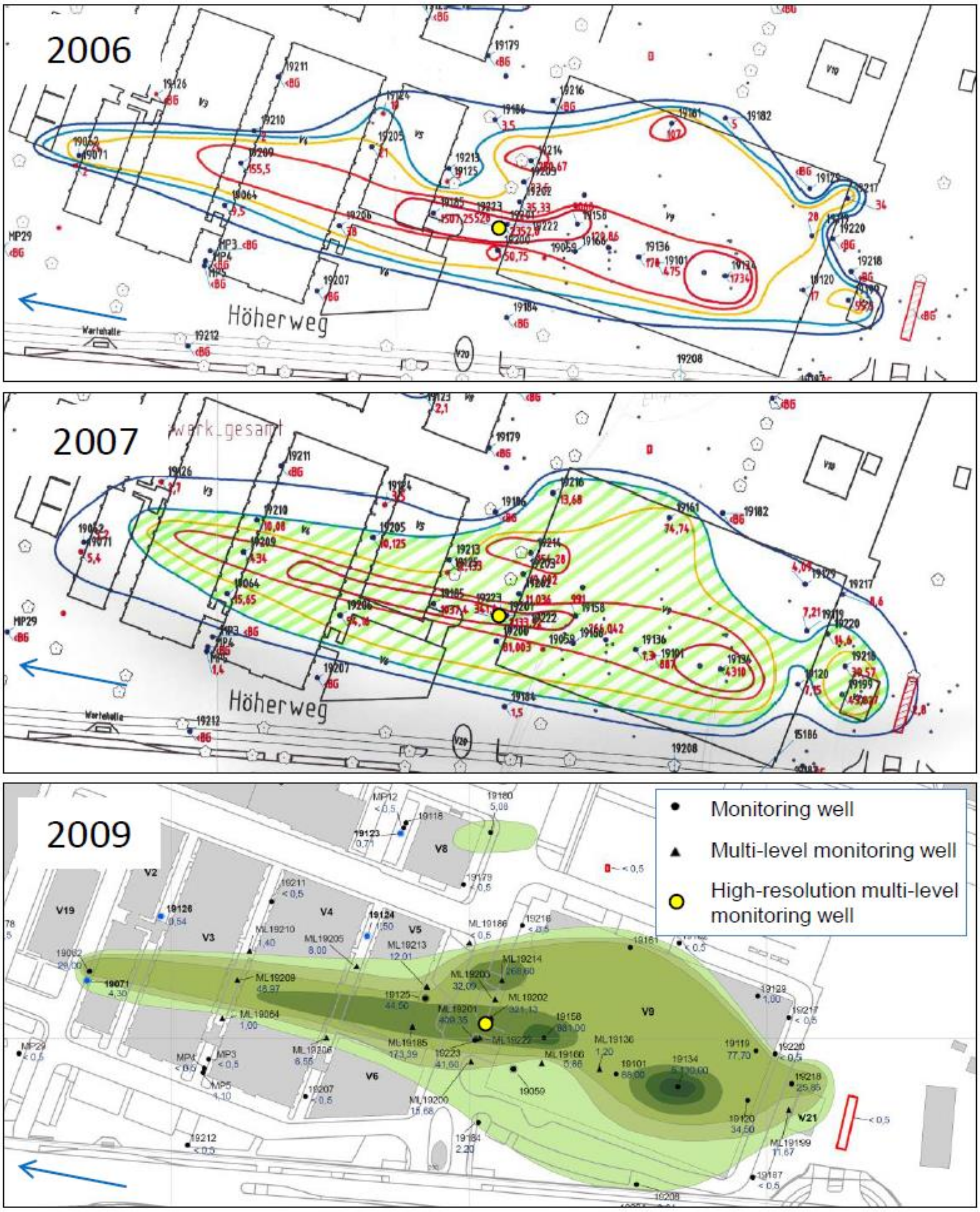

Fig. S1. Localization and horizontal extent of the toluene plume at the Flingern site over time. Total BTEX concentrations [mg l^{-1}] are given in numbers next to each well. The central localization of the HR-MLW within the lateral and longitudinal plume transect is illustrated by interpolation of total BTEX concentrations measured by the site owner (Stadtwerke Düsseldorf, SWD) via an extended network of conventional and regular multi-level monitoring wells. Kriging-interpolation for 2006 and 07 was done by the SWD, and by BFM-Umwelt (Munich, Germany) as contractor of the SWD for 2009. Well codes are given in black, total BTEX concentrations (µg l^{-1}) in red or blue. The main groundwater flow direction from ESE to WNW is indicated by arrows. <BG, below detection limit of 0.5 µg l^{-1}.

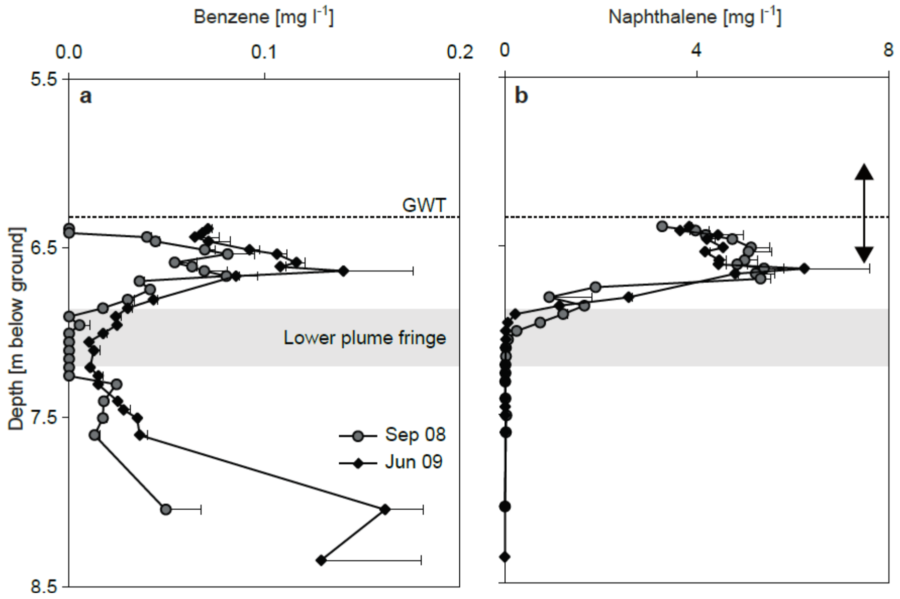

Fig. S2. Depth profiles of (**a**) benzene and (**b**) naphthalene concentrations in groundwater of the Flingern aquifer in Sep. 08 and Jun 09. Error bars indicate standard deviation (positive only) of measurements from duplicate samples. Further details are as in Fig. 2.

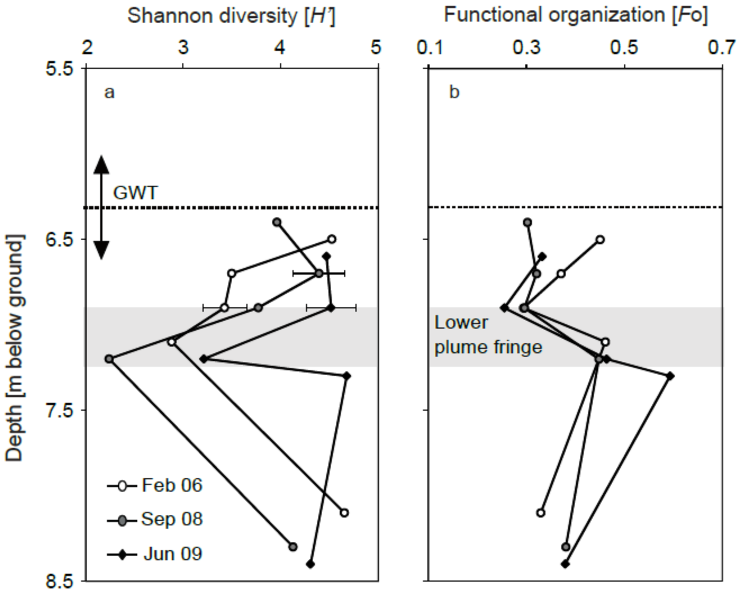

Fig. S3. Diversity measures of depth-resolved amplicon sequencing libraries of bacterial 16S rRNA gene fragments. Indices were calculated from rarefied (n = 1200) amplicon libraries per sample. Results from one selected set of replicate libraries from the lower plume core have been reported previously (35). Further details are as in Fig. 2.

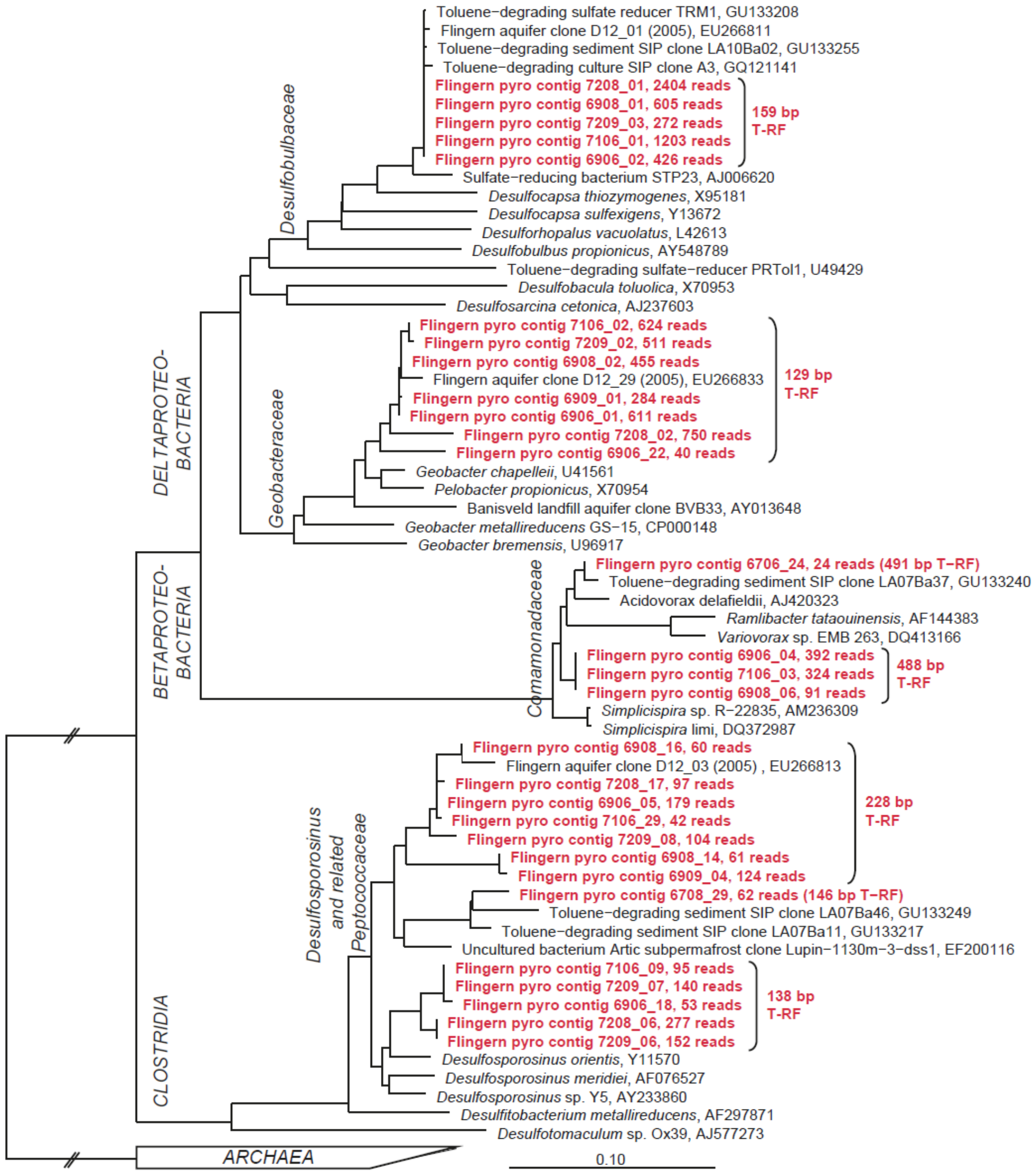

Fig. S4. Phylogenetic affiliation of selected assembled pyrosequencing contigs of ~520 bp bacterial 16S rRNA gene fragments retrieved from different depths of the Flingern aquifer. The tree was reconstructed as described (34), using full-length reference sequences and subsequent addition of shorter ones. Contig naming indicates depth (e.g. 7.1) and year (e.g. 09) of sampling, as well as contig number and comprised reads. Characteristic T-RFs for contigs and GenBank accession numbers of references are indicated. The scale bar represents 10% sequence divergence. Branch length to outgroup has been scaled down to 25%.

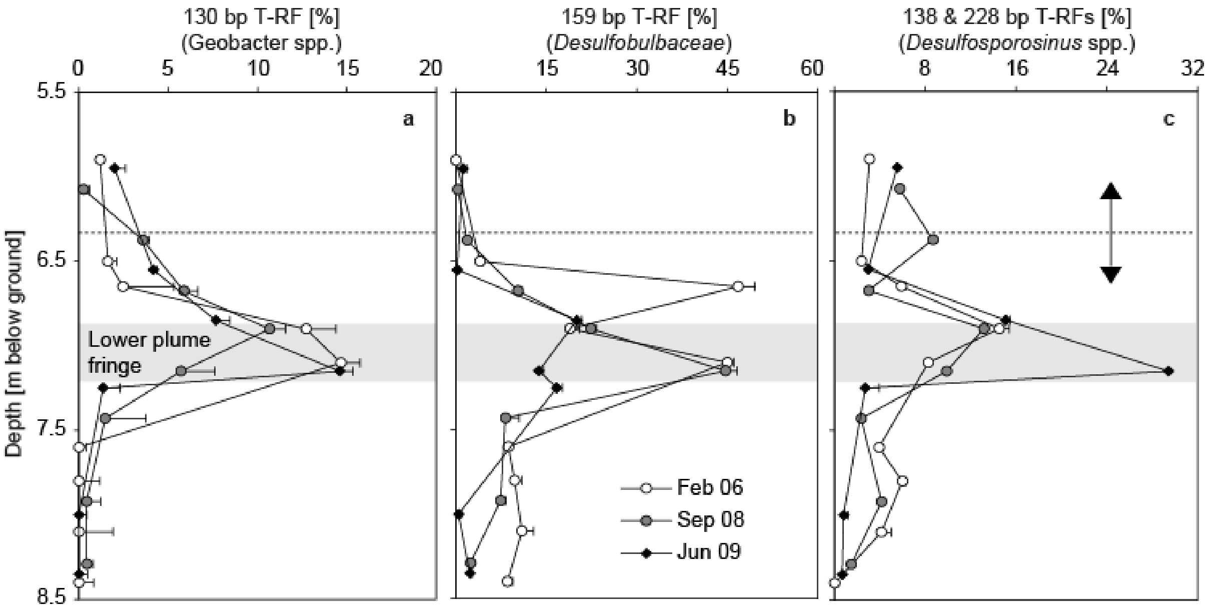

Fig. S5. Depth-distribution of relative T-RF abundance of characteristic degrader populations in replicate T-RFLP fingerprinting per sediment depth and time point. T-RF abundances are averaged abundances from triplicate sediment DNA extracts + standard error (positive only). Further details are as in Fig. 2.
